# Ruddlesden–Popper Oxyfluorides La_2_Ni_1–*x*_Cu_*x*_O_3_F_2_ (0 ≤ *x* ≤ 1): Impact of the Ni/Cu Ratio on the Thermal Stability and Magnetic Properties

**DOI:** 10.1021/acs.inorgchem.4c01330

**Published:** 2024-06-03

**Authors:** Jonas Jacobs, Hai-Chen Wang, Miguel A. L. Marques, Stefan G. Ebbinghaus

**Affiliations:** † Faculty of Natural Sciences II, Institute of Chemistry, Inorganic Chemistry, Martin Luther University Halle-Wittenberg, Kurt-Mothes-Straße 2, D-06120 Halle, Germany; ‡ Research Center Future Energy Materials and Systems of the University Alliance Ruhr, Faculty of Mechanical Engineering, Ruhr University Bochum, Universitätsstraße 150, D-44801 Bochum, Germany

## Abstract

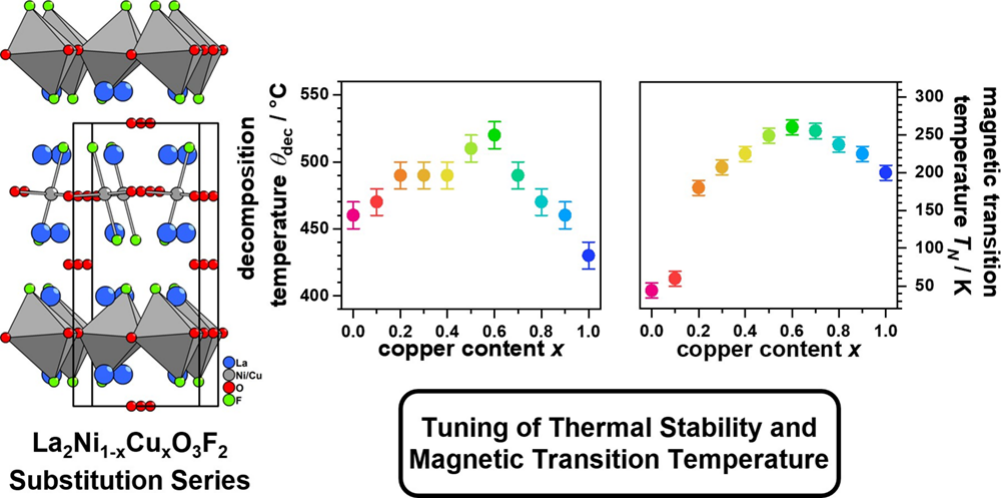

Ruddlesden–Popper oxyfluorides of the substitution series La_2_Ni_1–*x*_Cu_*x*_O_3_F_2_ (0 ≤ *x* ≤ 1) were obtained by topochemical fluorination with polyvinylidene fluoride (PVDF) of oxide precursors La_2_Ni_1–*x*_Cu_*x*_O_4_. The thermal stability and the temperature-dependent unit cell evolution of the oxyfluorides were investigated by high-temperature XRD measurements. The oxyfluoride with *x* = 0.6 shows the highest decomposition temperature of *θ*_dec_ ∼ 520 °C, which is significantly higher than the ones found for the end members La_2_NiO_3_F_2_ (*x* = 0) *θ*_dec_ ∼ 460 °C and La_2_CuO_3_F_2_ (*x* = 1) *θ*_dec_ ∼ 430 °C. The magnetic properties of all La_2_Ni_1–*x*_Cu_*x*_O_3_F_2_ oxyfluorides were characterized by field- and temperature-dependent measurements as well as DFT calculations of the magnetic ground state. An antiferromagnetic ordering was derived for all substitution levels. For the Néel temperature (*T*_N_), a nonlinear dependence on the copper content was found, and comparably high values of *T*_N_ in the region of 200–250 K were observed in the broad composition range of 0.3 ≤ *x* ≤ 0.8.

## Abstract 2

The thermal stability and magnetic properties of the Ruddlesden−Popper oxyfluorides La_2_Ni_1-*x*_Cu_*x*_O_3_F_2_ were investigated. As a result, tuning of the thermal stability and magnetic transition temperature was found to be possible by Ni/Cu substitution in a wide temperature range.

## Introduction

Transition metal oxyfluorides have attracted increasing attention due to their structural diversity and useful physical properties like F-ion conductivity,^1−3^ second harmonic generation,^4^ and superconductivity.^6−8^ Among these compounds, the Ruddlesden–Popper (RP) phases stand out as a fascinating class of materials characterized by a layered perovskite-like structure. This structure is composed of rock-salt (AX) layers alternating with perovskite blocks (ABX_3_) of thickness *n* and is therefore often written as (AX)(ABX_3_)_*n*_.

For the *n* = 1 case, the structure with the highest symmetry (*I*4*/mmm*) is referred to as K_2_NiF_4_ structure. Here, two distinguishable crystallographic sites exist for the anions ((1/2, 0, 0) and (0, 0, ≈ 0.16)) and one for the A- and B-type cations ((0, 0, ≈ 0.35) and (0, 0, 0), respectively). One additional, usually unoccupied third anion site is located within the AX building block (0, 1/2, and 1/4), which provides space for up to two additional anions per formula unit. The existence of this additional anion site causes high flexibility regarding the anion stoichiometry and structural details of RP compounds. This is even further enhanced when cations with more than one stable oxidation state are involved, which is primarily observed for transition metal B-cations. RP compounds are, therefore, an ideal platform for the exploration of various physical properties through controlled substitutions of cations and anions, yielding materials with potential applications, for example, in catalysis,^9,10^ energy storage,^11,12^ and electronic devices.^13,14^

The magnetic characterization of materials is one main area in solid state research, in particular, since the first discovery of nonmetal superconductivity in La_2–*x*_Ba_*x*_CuO_4–*y*_.^15^ Lately, *n* = 1 RP nickelates containing Ni^1+^ with T’-type RP structure are discussed as potential superconductors due to their d^9^ electron configuration, which is isoelectronic to the well-known cuprate superconductors.^16,17^ One first layered RP nickelate with T’-structure is La_2_NiO_3_F, which is obtained from reacting La_2_NiO_3_F_2_ with NaH or CaH_2_.^18,19^ The parent oxyfluoride La_2_NiO_3_F_2_ is an antiferromagnet below *T*_N_ = 49 K according to magnetization measurements and neutron powder diffraction studies.^18^ This antiferromagnetic spin arrangement was also found for the strongly structural-related, copper-containing compounds La_2_Ni_0.2_Cu_0.8_O_3_F_2_ and La_2_CuO_3_F_2_.^20^

Understanding the thermal stability of RP oxyfluorides is an additional important aspect for their application in high-temperature environments or as potential candidates for solid-state devices. Little has been published regarding the thermal stability of new RP oxyfluorides. On the other hand, such data are especially important as complex oxyfluorides are often found to be metastable due to the tendency to decompose, leading to the formation of thermodynamically stable binary fluorides like SrF_2_ and LaF_3_ or ternary oxyfluorides (i.e., LnOF). This is why oxyfluoride synthesis is recently often realized by low-temperature topochemical fluorination, for example, by reactions with fluoropolymers at their decomposition temperature.^21,22^ By this approach, several compounds like the metastable oxyfluoride La_2_NiO_2.5_F_3_ have been obtained.^23^

**Figure 1:**
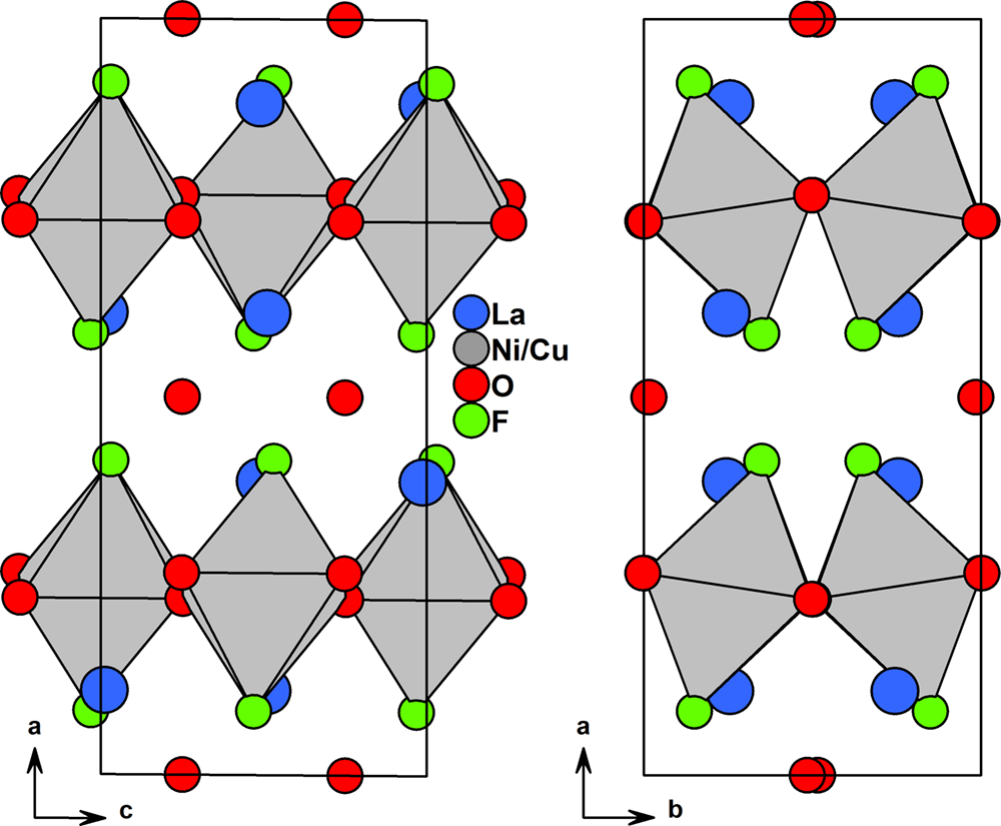
Crystal structure of La_2_Ni_0.5_Cu_0.5_O_3_F_2_ which crystallizes in space group *C*2*/c*. Projections of the *ac* and *ab* planes are shown.

The substitution of different transition metal cations within the oxyfluoride structure can have a strong impact on the local arrangement of oxygen and fluorine ions, influencing the structural distortions and stability, as well as the physical properties. In a previous article, we discussed how the replacement of Ni with Cu in La_2_Ni_1–*x*_Cu_*x*_O_3_F_2_ affects the crystal structure,^24^ and we found that the anionic ordering observed for La_2_NiO_3_F_2_^25^ persists throughout the substitution series. On the other hand, incorporation of the Jahn–Teller active Cu^2+^ results in a symmetry lowering from orthorhombic (*Cccm*; *x* ≤ 0.1) to monoclinic (*C*2*/c*; 0.2 ≤ *x* ≤ 0.9) and finally to triclinic (*P*1; *x* = 1.0) symmetry. The structure of *x* = 0.5 is shown in Figure 1, visualizing the unit cell dimensions and octahedral tilts.

In this study, we focus on the changes in thermal stability and magnetic properties resulting from the Ni/Cu substitution. For this, we have applied high-temperature X-ray diffraction (XRD) investigations as well as temperature- and field-dependent magnetization measurements. The magnetic investigations are complemented by DFT calculations yielding information on the magnetic ground states.

## Experimental Section

### Synthesis

The oxyfluorides La_2_Ni_1–*x*_Cu_*x*_O_3_F_2_ (in steps of *x* = 0.1) were synthesized as reported before.^24^ Fluorination was achieved by mixing the precursor oxides with polyvinylidene fluoride ((PVDF/CH_2_CF_2_)_n_) (Alfa Aesar) in a molar ratio of 1:1.05 (oxide: CH_2_CF_2_). The mixtures were slowly (2 K/min) heated to 350 °C, kept at this temperature for 48 h, and afterward allowed to cool down to room temperature in the box furnace.

### Characterization

HT-XRD patterns were recorded in transmission geometry on a STOE STADI-MP diffractometer operating with monochromatic Mo–Kα*1* radiation and equipped with a DECTRIS MYTHEN 1K detector and a capillary furnace (STOE HT1). The samples were filled in 0.5 mm diameter glass capillaries (Hilgenberg, glass No. 50) and were stepwise heated to 650 °C at a rate of 25 °C/min with isothermal intervals every 50 °C in the range of 50–300 °C and every 10 °C between 300 and 650 °C. In a typical experiment, at each isothermal step, an XRD pattern was recorded in the 2θ range 10–45° with 3 min acquisition time. For Rietveld refinements of the structural evolution based on the *in situ* XRD data, GSAS II^26^ was used. The instrumental resolution parameters were obtained from a LaB_6_ reference scan.

The magnetic characterization was performed with the ACMS magnetometer option of a Quantum Design Physical Property Measurement System (PPMS-9). Powder samples were loaded into gelatin capsules to minimize diamagnetic contributions. The temperature-dependent magnetic moment was measured in the range of 5–350 K in the DC mode for external fields from 0.01 to 9 T applying zero-field-cooled (ZFC) and field-cooled (FC) conditions. Prior to the scans, the superconducting magnet was set from 2 T to zero in the oscillating mode to reduce the trapped flux. Field-dependent measurements were performed at 5 K, and full hysteresis loops were recorded in the range from 9 to −9T.

DFT calculations were performed using the projector augmented wave (PAW) approach^27,28^ implemented in the Vienna ab initio simulation (VASP) package. For structural optimization, uniform Γ-centered k-point grids with a density of 2000 k-points per reciprocal atom were used to sample the Brillouin zone. Denser k-grids with a density of 5 k-points/Å^–1^ (∼4000 k-points per reciprocal atom) were applied for calculations of energy differences between different magnetic orderings. The cutoff for the plane-wave basis was set to 520 eV, and the total energies were converged to less than 0.01 meV/cell. The Perdew–Burke–Ernzerhof^29^ exchange–correlation functional with an on-site 6.2 eV repulsive *U* correction was used for Ni 3d-states. For the compositions containing both Ni and Cu with possible cationic disordering, different magnetic and structural configurations were generated using the cluster expansion method as implemented in the ATAT^30^ package.

## Results and Discussion

### Thermal Stability

In a first attempt, we tried to study the thermal stability of the oxyfluorides by combined thermogravimetric differential thermal analysis (TGA/DTA) measurements (shown for *x* = 0.3 in Figure S1) with coupled mass spectrometry (MS). Heating in air up to 1200 °C resulted in mixtures of LaOF and the binary oxides NiO/CuO as final decomposition products (identified by XRD), and no significant change was found for either the mass, the DTA, or the MS signals of H_2_O, CO_2_, HF, F, or O_2_ in the whole temperature range. The decomposition of La_2_(Ni/Cu)O_3_F_2_ with LaOF and (Ni/Cu)O as decomposition products is expected to occur without a mass change. The absence of a mass change in the TGA data and the fact that no volatile species containing F or a decrease in O_2_ in the reaction atmosphere was observed in the MS was seen as additional confirmation of the oxyfluorides O/F stoichiometry. These results also underline the absence of carbon-related species in the synthesized oxyfluorides, as these would result in the formation of CO or CO_2_, which were also not observed by mass spectrometry. Without the presence of a mass change and with only very broad and weak changes in the DTA signal, the determination of decomposition temperature is not possible by thermal analysis.

**Figure 2:**
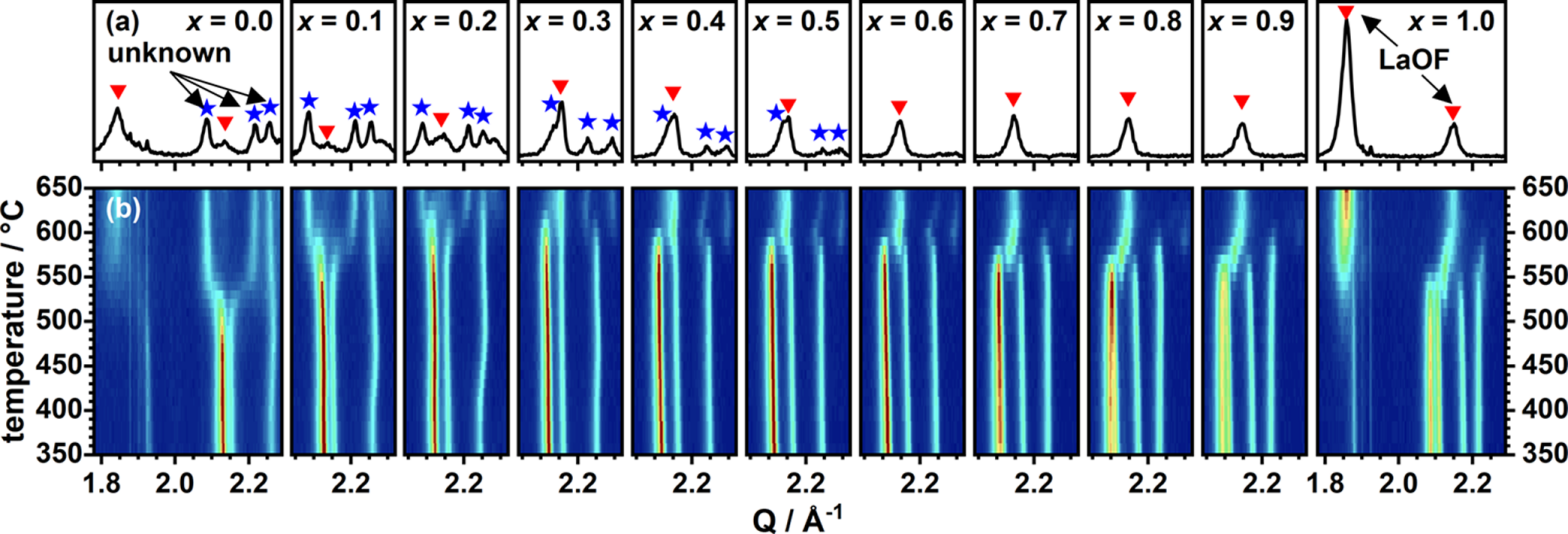
XRD patterns collected at 650° (a) and contour plots of the temperature-dependent (350–650 °C) XRD patterns (b) both in the region of the main reflections ((311), (31–1), (020), and (002)). In (a), reflections of LaOF are marked by red triangles, and the ones of an unidentified decomposition product are marked with blue asterisks.

The thermal stability was therefore evaluated by temperature-dependent XRD measurements. The diffraction patterns in the region of the most intense reflections are plotted in Figure 2. Surprisingly, heating the oxyfluorides to 650 °C results in the formation of different decomposition products depending on the Cu-content *x* (see Figure 2a). For values of *x* > 0.6, the formation of LaOF (marked with red triangles) as the crystalline fraction and an amorphization of the Ni/Cu-fraction take place as has been previously reported for the decomposition of La_2_NiO_3_F_2_^25^ and La_2_CuO_3_F_2_.^20^ For the lower Cu contents with *x* ≤ 0.6, an additional crystalline phase of unknown composition (marked with blue stars for *x* = 0–0.5) is found, which was not described before. These additional signals most probably belong to a less orthorhombically distorted K_2_NiF_4_-like structure with an increased longest axis. This assumption is based on the shift of the most intense peak ((311), in the *Cccm* unit cell of the oxyfluoride) to lower *Q* values, indicating an increase of *a*, while (020) and (002) shift to higher values, giving rise to a decreased orthorhombic distortion. The fraction of this phase in the diffraction patterns at 650 °C is the highest in *x* = 0.1 and decreases with increasing *x*. Attempts to isolate this unknown phase were not successful yet, and the structure of this decomposition product, therefore, remains unknown.

**Figure 3:**
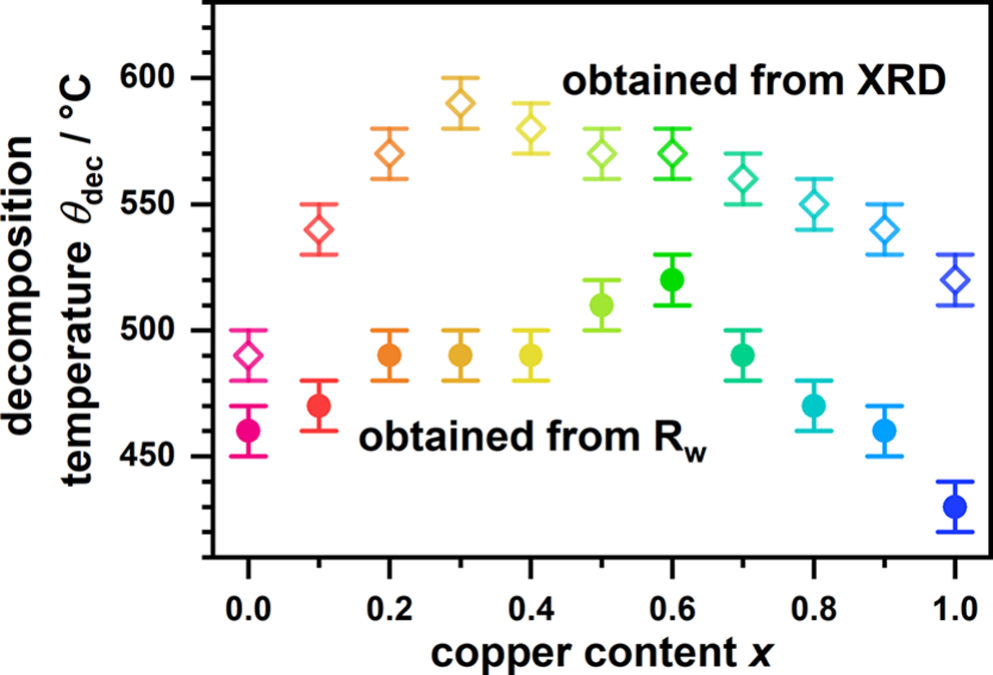
Decomposition temperature *θ*_dec_ of La_2_Ni_1–*x*_Cu_*x*_O_3_F_2_ in dependence of the Cu-content *x* as obtained directly from the XRD data (open symbols) and from the *R*_w_ value obtained by Rietveld refinements (closed symbols).

Based on the intensity evolution of the main reflections in Figure 2b, the compound with *x* = 0.3 is by far the most stable one. For this compound, the most prominent peaks of the oxyfluorides are still clearly present even at 600 °C. Here, a highly increased thermal stability is found, which decreases toward both sides of the substitution series. When taking into account that the decomposition involves the formation of LaOF, the decomposition temperature, *θ*_dec_, needs to be significantly lowered. For *x* = 0.3, first visible signals of LaOF already appear at ∼500 °C. Two sets of *θ*_dec_ values are, therefore, plotted in Figure 3. The first set *θ*_dec_ was defined as the last temperature step where the signals of the oxyfluorides are still present (open symbols, labeled as *obtained from XRD*). The second set of *θ*_dec_ values was determined as the temperature step where a sudden significant increase in the *R*_w_ value (*R*_w_ ≈ 10% → 15%) of the structural refinement was found. This approach was chosen as *R*_w_ tends to be the most sensitive to the formation of additional reflections (e.g., due to LaOF formation) or peak broadening (when keeping all shape parameters fixed during the refinements). By this approach, we obtain significantly lower but more realistic *θ*_dec_ values. Based on this second approach the highest thermal stability is found for the *x* = 0.6 compound with a decomposition temperature of ∼520 °C. The thermal stability of the oxyfluorides La_2_Ni_1–*x*_Cu_*x*_O_3_F_2_ decreases almost linearly toward both end members and was determined as ∼460 °C for La_2_NiO_3_F_2_ and ∼430 °C for La_2_CuO_3_F_2_. The increase of the thermal stability toward the middle of the substitution series might be explained by entropy stabilization due to random mixing to the Ni/Cu sites. This should be further investigated by performing fluorination experiments with oxide precursors containing three or more *B*-cations in equal amounts. By Ni/Cu substitution, an effective way was found to increase the thermal stability of the 2F oxyfluorides by almost 100 °C while retaining the overall structural distortion.

**Figure 4:**
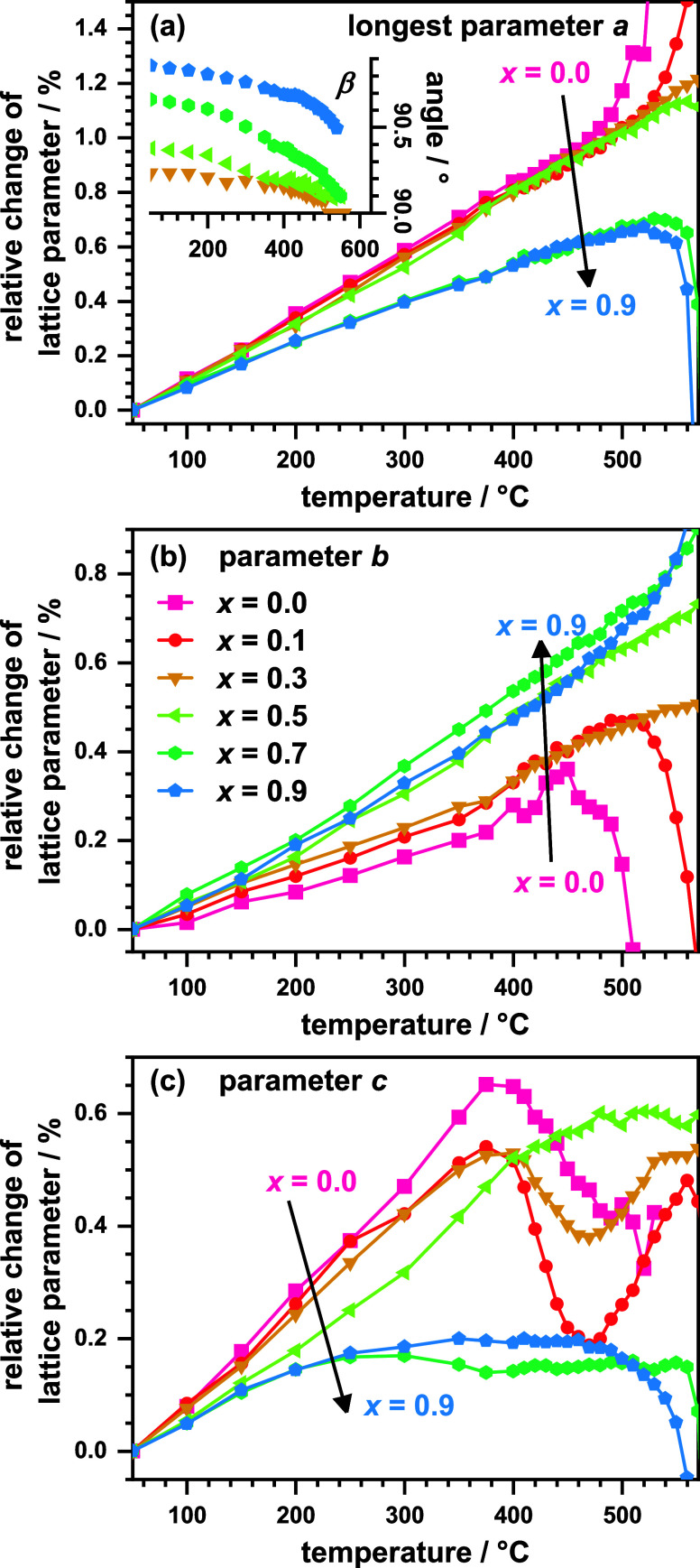
Relative change of the lattice parameters *a* (a), *b* (b), and *c* (c) and the angle β (inset in (a)) as a function of temperature plotted for the samples with *x* = 0.0, 0.1, 0.3, 0.5, 0.7, and 0.9.

The thermal evolution of the unit cell parameters was quantified by Rietveld refinements. The temperature-dependent values of *a*, *b*, and *c* (relative to values observed at 50 °C) as well as the monoclinic angle *β* are shown in Figure 4a–c. The data for *x* = 1 are not shown as this compound crystallizes in a triclinic unit cell, and its thermal evolution was already discussed in our previous paper.^20^ The longest axis *a* and the second longest axis *b* both increase linearly with temperature up to the decomposition temperature *θ*_dec_. The unit cell expansion is strongly anisotropic, as the relative increase in *b* is about half as large as the increase of *a*. The thermal expansion, therefore, happens primarily perpendicular to and not within the perovskite layers. Similar results were already found by Wissel et al. for La_2_NiO_3_F_2_ (*x* = 0).^25^ The monoclinic unit cell distortion, reflected by the monoclinic angle *β*, is also strongly reduced with increasing temperature. For *x* = 0.2–0.6 (plotted as inset in Figure 4a), *β* reaches 90° within the error of determination before the thermal decomposition starts. A phase transition at elevated temperatures from monoclinic to orthorhombic seems likely for these compositions, resulting from the anisotropic thermal unit cell expansion. This transition is reversible as found by the temperature-dependent *in situ* XRD data, which is shown for the *x* = 0.6 compound in the supplement (Figure S2).

For the second orthorhombic axis *c*, the linear increase is also observed up to ∼400 °C. Above this temperature, strong deviations from the linear behavior are observed, which are especially pronounced for the compounds with *x* ≤ 0.3. This points to a second phase transition happening before decomposition. To check for a reversible phase transition, a sample with *x* = 0.2 was heated to 470 °C twice with intermediate cooling to 350 °C (temperature-dependent XRD patterns from this experiment are shown in the supplement (Figure S3)). From this experiment, the observed anisotropic decrease of *c* above 400 °C was found to be irreversible. Based on the TGA/DTA-MS data shown in Figure S1, no evolution of H_2_O was observed in this temperature region. The release of potentially co-incorporated H_2_O is, therefore, excluded as a possible explanation. A more suitable explanation for such a behavior might be a structural reorientation in the unit cell, which could be caused by healing of anionic defects or partial (re)oxidation of Ni or Cu. This is possible as the experiments were carried out in capillaries, which were open to air. To further elucidate this behavior, *in situ* neutron diffraction experiments are planned to gain deeper insights into the occupation of the anionic positions because deviations in the interlayer occupation may result in such a behavior.

### Magnetic Properties

Upon substitution of Ni^2+^ (3d^8^ electron configuration) with Cu^2+^ (3d^9^ configuration), a strong impact on the magnetic properties of the solid solution oxyfluorides is expected. One reason is that in the case of Ni^2+^, only AFM super exchange interactions are expected according to the Goodenough–Kanamori–Anderson rules. By incorporation of Cu^2+^ at Ni^2+^ positions, AFM super exchange interactions are expected between Cu^2+^ centers, and additional weak ferromagnetic interactions of half occupied Ni-d-orbitals and occupied Cu-d-orbitals are expected based on the GKA rules. An additional Jahn–Teller elongation of the Cu-containing octahedra was derived from our previous structural investigations.^24^ This elongation was additionally found to yield different octahedral tilting components depending on x. As a result, altered angles for the longer ranged Ni/Cu–F_ap_-F_ap_-Ni/Cu interaction of neighboring octahedral layers are also found; differing super exchange interactions between the perovskite layers, therefore, seem likely.

**Figure 5:**
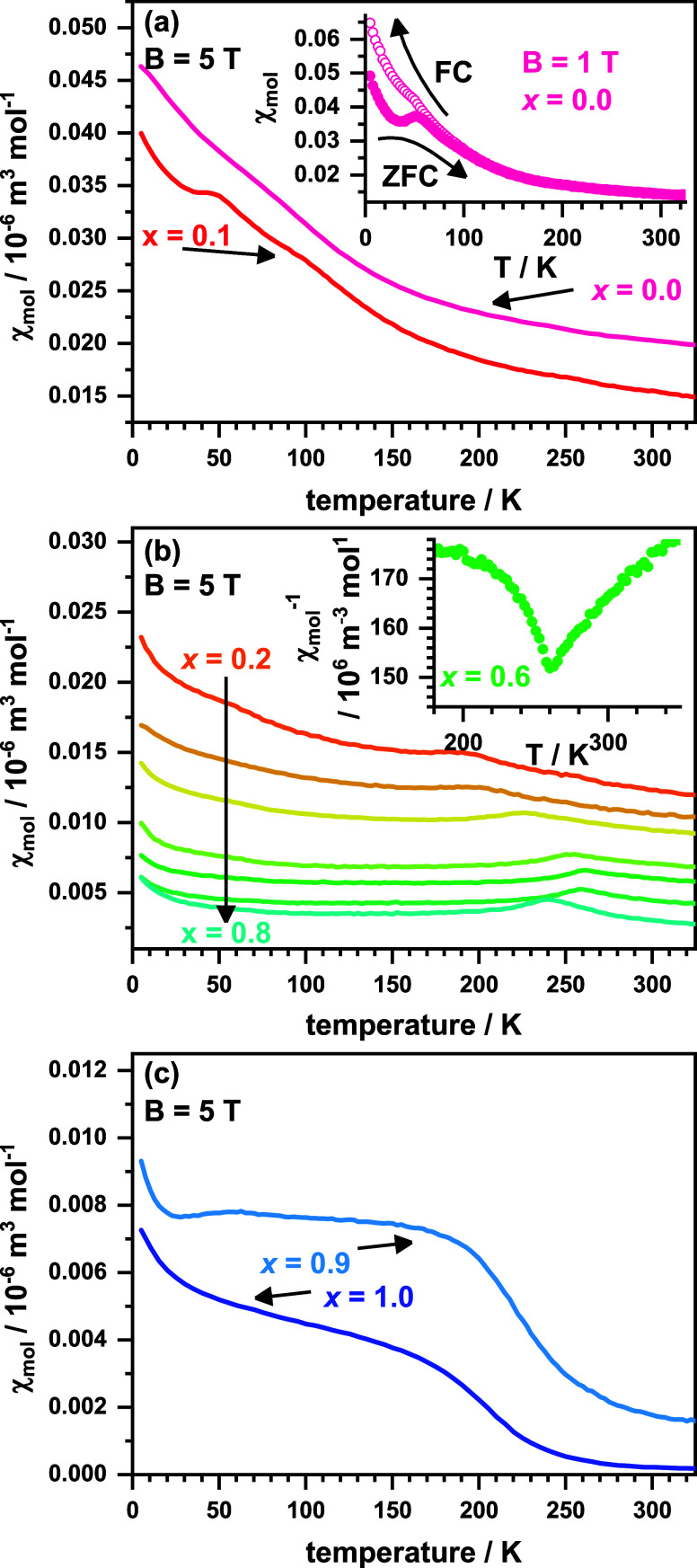
Susceptibility versus temperature at 5 T for La_2_Ni_1–*x*_Cu_*x*_O_3_F_2_ with *x* = 0.0 and 0.1 (a), 0.2–0.8 (b), and 0.9, 1.0 (c). The inset in (a) shows the 1 T data for *x* = 0.0. The inset in (b) shows the inverse susceptibility of *x* = 0.6.

To study the magnetic properties, temperature-dependent magnetization measurements were performed between 5 and 350 K in an external field of 5 T for all samples. The resulting *χ*_mol_ vs *T* curves are plotted in Figure 5a–c. All compounds show signs of weak paramagnetism and considering the overall shape of the *χ*_mol_ vs *T* data, three different general curve shapes are obtained, which will be discussed in the following. The susceptibility data of the samples with *x* = 0.0 and 0.1 exhibit a similar appearance with the *x* = 0.1 data being shifted to generally lower *χ*_mol_ values. This reflects a decreasing number of unpaired electrons due to the increasing copper content *x*. An additional cusp below ∼60 K in the *x* = 0.1 data hints at a magnetic transition for this compound. A very similar feature was previously reported for La_2_NiO_3_F_2_ at 49 K (measured at 1 T) and was interpreted as the transition from a paramagnetic to an antiferromagnetic spin arrangement. This model was supported by low-temperature neutron powder diffraction data.^18,25^ The same paramagnetic to antiferromagnetic transition can be assumed for the *x* = 0.1 compound. Interestingly, in our data, no peak or cusp was observed in the susceptibility data around 50 K for La_2_NiO_3_F_2_ (*x* = 0.0). The cusp is most probably suppressed by the high applied field (5 T vs 1 T used in the literature). In fact, additional measurements in a significantly lower field of 1 T (plotted as inset in Figure 5a) also show this feature for *x* = 0.0 compound accompanied by a ZFC/FC splitting of the data, which was not observed in high fields.

The fact that the *x* = 0.0 and 0.1 compounds possess a similar behavior is in concordance with the similar crystallographic structure as both compounds crystallize in space group *Cccm*. The 0.2 ≤ *x* ≤ 0.8 compounds of the substitution series all possess a *χ*_mol_ vs *T* behavior, which in its overall shape is very similar to the antiferromagnetic behavior of the *x* = 0.8 compound La_2_Ni_0.2_Cu_0.8_O_3_F_2_ as previously described.^20^ All compounds exhibit a relatively large temperature-independent contribution, which decreases almost linearly with increasing copper content *x* from 0.015 × 10^–6^ m^3^ mol^–1^ (*x* = 0.2; at 325 K) to 0.004 × 10^–6^ m^3^ mol^–1^ (*x* = 0.8). This most probably resembles the decrease in unpaired electrons caused by Ni/Cu substitution. In the region of 205–260 K, broad peaks or cusps are found. These are interpreted as the sign of a paramagnetic to an antiferromagnetic transition. In contrast, the *x* = 0.9 and 1.0 compounds both show a clearly visible step-like increase of the overall very low magnetic susceptibility below *T*_C_ = 220 K (*x* = 0.9) and 190 K (*x* = 1.0). This behavior is the sign of a weak uncompensated moment that we recently interpreted as ferrimagnetic contribution for La_2_CuO_3_F_2_ due to a frustrated AFM spin arrangement.^20^ The same ferrimagnetic contribution is assumed for *x* = 0.9. The highly similar magnetic behavior of *x* = 0.9 and 1.0 can also be seen as a hint to a similar crystallographic structure. High-resolution synchrotron XRD data might confirm a triclinic unit cell for *x* = 0.9 in future studies.

**Figure 6:**
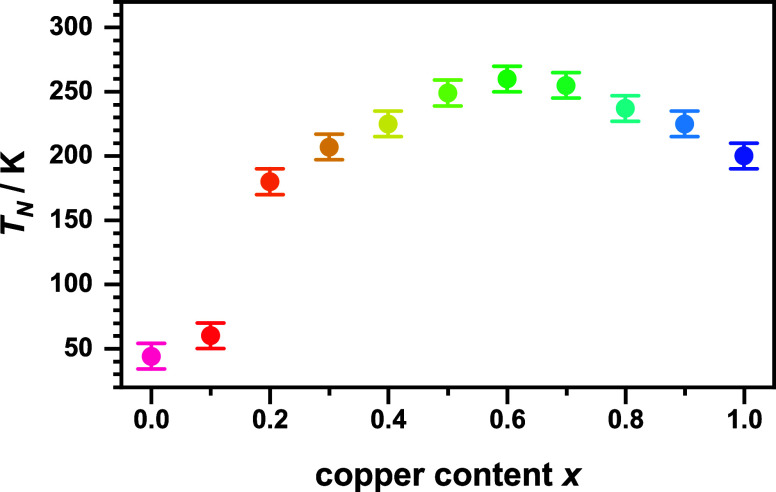
Néel temperature *T*_N_ of the oxyfluorides La_2_Ni_1–*x*_Cu_*x*_O_3_F_2_ as a function of the Cu-content *x*. The values were obtained as maxima from d*χ*_mol_*/*d*T* vs *T* plots.

The transition temperatures (Néel temperature; *T*_N_) were obtained as maxima from d*χ*_mol_*/*d*T* vs *T* plots. The *T*_N_ values are plotted in Figure 6. It has to be noted that Ni/Cu substitution results in a strong increase of *T*_N_ toward the middle of the substitution series, which is similar to the increased thermal stability. Values in the range 50–260 K are obtained with *x* = 0.6 exhibiting the highest antiferromagnetic ordering temperature.

To further distinguish between ferro- and antiferromagnetic spin arrangements (note that ferrimagnetism also is the result of an “imperfect” AFM spin arrangement), the sign of the Weiss constant is commonly used, which in principle can be extracted by fitting the high-temperature region of *χ*_mol_^–1^ vs *T* data with the Curie–Weiss law. Unfortunately, a linear behavior of the inverse susceptibility data was observed only for *x* = 0.0 and 0.1. The linear Curie–Weiss fits of these two data sets in the region of 250–325 K are presented in the supplement (Figure S4). For both compounds, similar paramagnetic moments of 2.60 and 2.50 μ_B_ (*x* = 0.0 and 0.1, respectively) are obtained. Both values are slightly lower than the expected spin-only moments of 2.83 μ_B_ for *x* = 0 and 2.74 μ_B_ for *x* = 0.1. This is not surprising because for both ions crystal field effects need to be taken into account. While in a (basically) octahedral coordination, increased μ_B_ values are expected (as found, e.g., for Ni^2+^ in 6H-BaTiO_3_);^32^ the situation becomes more complex for crystal fields of lower symmetry. Furthermore, the underlying models are valid only for diluted magnetic systems, whereas strong interionic interactions occur in the materials studied here. A strongly negative Weiss constant of θ ≈ −400 K was obtained for both compounds; this is in agreement with the published antiferromagnetic spin arrangement of La_2_NiO_3_F_2_. For *x* = 0.1, a similar antiferromagnetic ordering with a slightly increased Néel temperature of 60 K was deduced. For the inverse susceptibility data of all other members of the substitution series, a nonlinear temperature dependence is found, which is shown as an inset in Figure 5b for *x* = 0.6. Such a behavior may, in principle, be fitted by the Curie–Weiss law with the addition of a temperature-independent component. Such fits are in the present case impeded by an insufficient number of data points that are available due to the rather high transition temperatures (in the region of 190–260 K). Therefore, no reliable information on the Weiss constants or the paramagnetic moments can be derived for the compounds with *x* ≥ 0.2.

DFT calculations of the magnetic configuration were performed for 32 atom unit cells in both possible space groups of La_2_Ni_1–*x*_Cu_*x*_O_3_F_2_ (*Cccm* and *C*2*/c*) with *x* = 0.0, 0.25, 0.5, 0.75, and 1.0. These calculations were used to obtain additional information on the type of magnetic interactions (i.e., ferromagnetic or antiferromagnetic). For the sake of simplicity, fluorine was assumed to solely occupy the apical positions in the octahedra, and the *P*1 symmetry of the *x* = 1.0 compound was not taken into account. In this 32-atom unit cell, there are two octahedral layers (denoted as A and B), and in each layer there are two octahedra. The first to fourth nearest magnetic interactions were considered with the first nearest interaction being between Ni/Cu in neighboring octahedra of the same layer (A1A2 and B3B4) and thus of antiferromagnetic nature. The interaction of the second nearest centers is considered for atoms on the same position in the same octahedral layer, but in the neighboring cells (e.g., A1A1 and B3B3) such interactions are expected to be ferromagnetic. The third and fourth nearest interactions were considered as weak interactions between Ni or Cu atoms of different octahedral layers (e.g., A1B4, A2B3). The ground states of different compositions and the energy difference with respect to nonmagnetic configurations are listed in Table 1. It was found that the antiferromagnetic ground states are lower in energy for all configurations in both space groups with the exception of the pure copper compound *x* = 1.0, where no magnetic ordering is found in *C*2*/c*_*.*_ This is partially in concordance with the experiment where very low susceptibility values are obtained for La_2_CuO_3_F_2_, but the small ferromagnetism at low temperatures is not found in these calculations. For all other compounds, higher AFM coupling strengths are found for the *C*2*/c* cell, and overall stronger magnetic interactions are found for the Ni-rich compounds. By these results, the antiferromagnetic ordering of all samples between 0.0 ≤ *x* ≤ 0.75 can be confirmed, even though the highest *T*_N_ values of *x* = 0.4–0.6 are not explained by the calculations.

**Table 1:** Calculated Ground State Magnetic Configurations for Two Phases (*Cccm* or *C*2/*c* Symmetry) for Different Ni/Cu Concentrations^a^

Cu-content*x*	*Cccm*				Δ*E* (meV/atom)
	A1	A2	B3	B4	
0	Ni (1.8)	Ni (−1.8)	Ni (−1.8)	Ni (1.8)	–173
0.25	Ni (1.7)	Cu (−0.5)	Ni (−1.8)	Ni (1.8)	–139
0.5	Ni (1.7)	Cu (−0.5)	Cu (0.5)	Ni (−1.7)	–107
0.75	Ni (1.7)	Cu (−0.5)	Cu (−0.3)	Cu (0.3)	–53
1	Cu (0.3)	Cu (−0.3)	Cu (0.3)	Cu (−0.3)	–2

	*C*2*/c*				
	A1	A2	B3	B4	
0	Ni (1.8)	Ni (−1.8)	Ni (−1.8)	Ni (1.8)	–176
0.25	Ni (1.8)	Ni (−1.8)	Cu (−0.5)	Ni (1.7)	–145
0.5	Cu (0)	Cu (0)	Ni (−1.8)	Ni (1.8)	–97
0.75	Cu (0)	Cu (0)	Cu (−0.5)	Ni (1.7)	–66
1	Cu (0)	Cu (0)	Cu (0)	Cu (0)	0

a For each ground state, the atom occupying the four different (A1–B4) octahedral centers is listed as well as its magnetic moment (in μ_B_). The last column shows the energy difference of the ground state with respect to the not magnetically ordered (paramagnetic) configuration.

**Figure 7:**
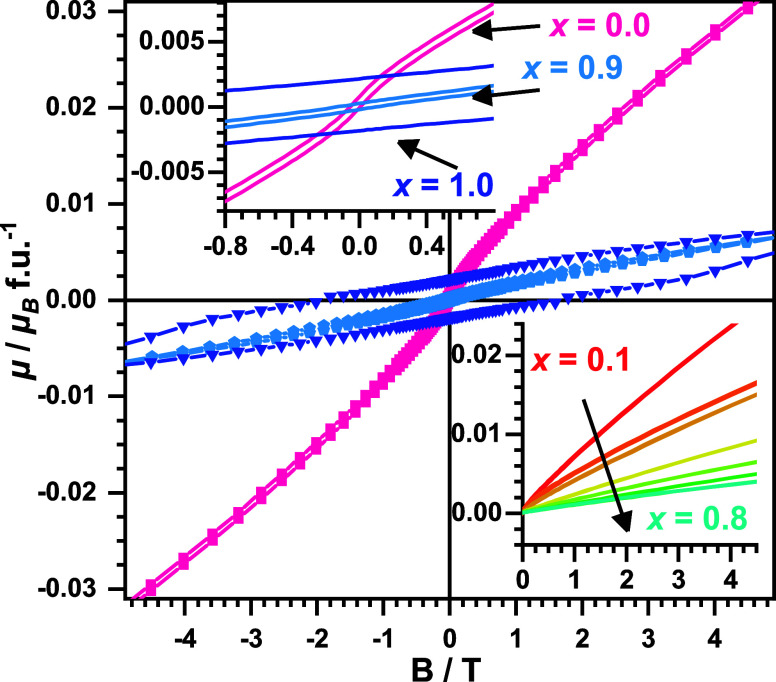
Magnetic moment (*μ*) vs field data for La_2_Ni_1–*x*_Cu_*x*_O_3_F_2_ obtained at 5K.

Field-dependent magnetization measurements (*μ* vs B) were carried out at 5 K for all oxyfluorides. The obtained data are plotted in Figure 7. A hysteretic behavior with very small saturation moments <0.01 μ_B_ f.u.^–1^ is found for the oxyfluorides with *x* = 0.0, 0.9, and 1.0. This finding is in agreement with a small macroscopic ferrimagnetic moment, which was already described for La_2_NiO_3_F_2_^18^ and La_2_CuO_3_F_2_^20^ to arise from an canted AFM spin arrangement. The same interpretation might be applied for *x* = 0.9, even though the hysteresis of this compound is rather weak with a coercivity of 0.12 T compared to 2 T obtained for *x* = 1.0 (La_2_CuO_3_F_2_). The presence of a magnetic hysteresis for the sample with *x* = 0.9 can be considered as an additional hint to a high structural similarity to the pure *x* = 1.0 compound. For all other compositions 0.1 ≤ *x* ≤ 0.8, no hysteresis or step-like behavior was found in the *μ* vs B data at 5K. Only very slightly sigmoidally shaped curves are found, which is consistent with the proposed antiferromagnetic ordering.

The temperature-dependent susceptibility data of the selected precursor La_2_Ni_1–*x*_Cu_*x*_O_4_ oxides (*x* = 0.0, 0.1, 0.3, 0.5, 0.7, 0.9, and 1.0) are plotted in the supplement for comparison (Figure S5). The oxides with 0.1 ≤ *x* ≤ 0.8 are paramagnetic, and no sign of magnetic transition is found above 10 K. For La_2_NiO_4_, a small step at 150 K indicates the PM to AFM transition, which is often reported in the literature with *T*_N_ strongly depending on the exact oxygen stoichiometry.^33−35^ For La_2_CuO_4_, only a very small temperature-independent susceptibility was found in agreement with previous investigations.^20,36^ We, therefore, want to emphasize that the magnetic behavior of the oxyfluorides is highly different compared to the ones of their parent oxides. Furthermore, Ni/Cu substitution is beneficial for archiving antiferromagnetic-ordered oxyfluorides with comparatively high ordering temperatures tunable in the range of 50–250 K.

## Conclusions

Topochemical fluorination of the solid solution La_2_Ni_1–*x*_Cu_*x*_O_4_ was carried out with PVDF as the fluorination agent, yielding phase pure oxyfluorides La_2_Ni_1–*x*_Cu_*x*_O_3_F_2_ for the whole substitution series 0 ≤ *x* ≤ 1. Temperature-dependent XRD experiments revealed a substantially increased thermal stability compared to both end members La_2_NiO_3_F_2_ and La_2_CuO_3_F_2_. The highest decomposition temperature of *θ*_dec_ ≈ 520 °C was found in the middle range for x = 0.6. The impact of the substitution of Ni by Cu on the magnetic properties of the oxyfluorides was studied by temperature- and field-dependent magnetization measurements. An antiferromagnetic ordering was obtained for all compounds with the Néel temperature varying in the range of 50–250 K. The AFM ground state was also confirmed by DFT calculations, performed in steps of *x* = 0.25. For the compounds with *x* = 0.0, 0.9, and 1.0, an additional week ferrimagnetic contribution to the field-dependent magnetization was attributed to an improper antiferromagnetic spin alignment, that is, canted antiferromagnetism. This interpretation needs to be confirmed by neutron powder diffraction experiments below *T*_N_.

## Supporting Information Available

TGA/DTA-MS data for the thermal decomposition of La_2_Ni_0.7_Cu_0.3_O_2_F_3;_ temperature dependent XRD-data for the tempering of La_2_Ni_0.4_Cu_0.6_O_3_F_2_; temperature dependent XRD-data and extracted lattice parameters for the heating of La_2_Ni_0.8_Cu_0.2_O_3_F_2;_ inverse susceptibility vs temperature data for *x* = 0.0 and 0.1 as well as the linear Curie–Weiss fits to the high temperature region; susceptibility vs temperature for La_2_Ni_1–*x*_Cu_*x*_O_4_ obtained in an external field of 1T (PDF)
