# Supplementary material for: Ruddlesden–Popper Oxyfluorides La2Ni1–xCuxO3F2 (0 ≤ x ≤ 1): Impact of the Ni/Cu Ratio on the Thermal Stability and Magnetic Properties
Source: Inorg Chem. 2024 Jun 3;63(24):11317–24. doi: 10.1021/acs.inorgchem.4c01330 (PMC11190993; doi:10.1021/acs.inorgchem.4c01330)
Supplement: Supplementary file 1 — ic4c01330_si_001.pdf [file ic4c01330_si_001.pdf]

Ruddlesden-Popper Oxyfluorides $La_2Ni_{1-x}Cu_xO_3F_2$ $(0 \leq x \leq 1)$: Impact of the Ni/Cu Ratio on the Thermal Stability and Magnetic Properties

Jonas Jacobs[*,†], Hai-Chen Wang[‡], Miguel A. L. Marques[‡], and Stefan G. Ebbinghaus[†]*

[†] Martin Luther University Halle-Wittenberg, Faculty of Natural Sciences II, Institute of Chemistry, Inorganic Chemistry, Kurt-Mothes-Straße 2, D-06120, Halle, Germany

[‡] Research Center Future Energy Materials and Systems of the University Alliance Ruhr, Faculty of Mechanical Engineering, Ruhr University Bochum, Universitätsstraße 150, D-44801 Bochum, Germany

Email: jonas.jacobs@chemie.uni-halle.de

Supporting Information

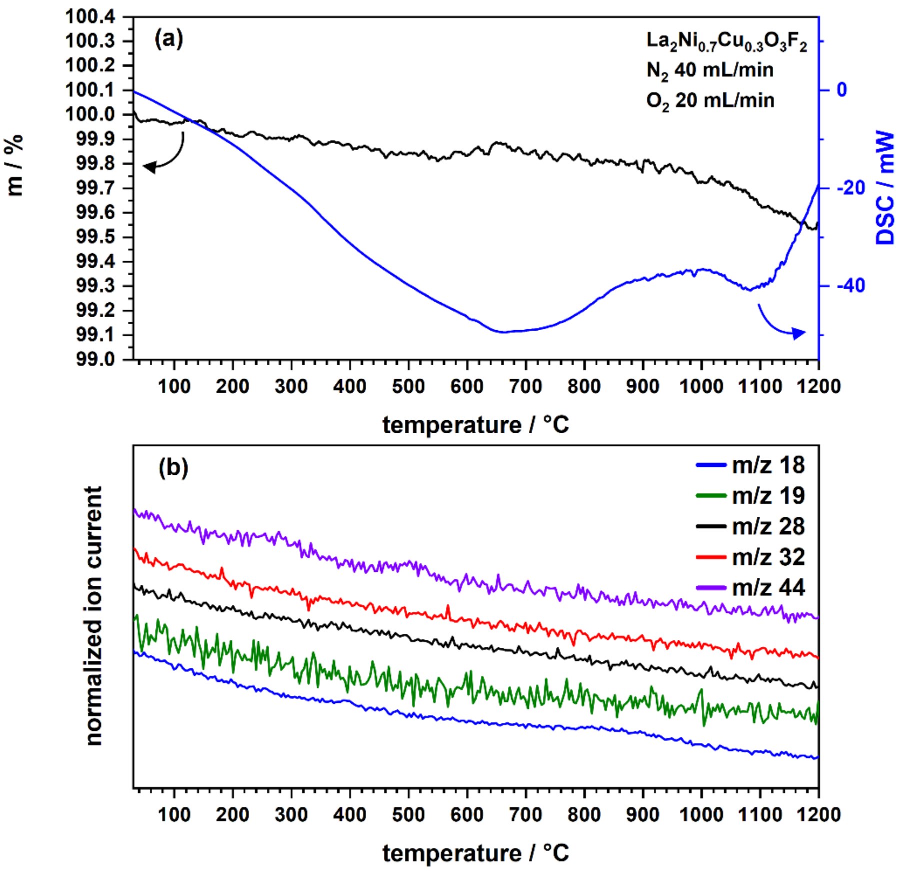

Figure S1: TGA/DTA-MS data for the thermal decomposition of $La_2Ni_{0.7}Cu_{0.3}O_3F_2$ obtained in flowing N_2/O_2. In (a) the temperature dependent mass change is shown in combination with the DSC signal. In (b) the normalized ion current is shown and the lines are shifted equally. The m/z ratios were selected to represent H_2O, F, N_2/(CO), O_2, and CO_2 as they are the most expected decomposition products. *The experiments were performed on a NETZSCH STA 449F5 Thermobalance with a coupled mass spectrometer (Pfeiffer Vacuum GSD 350 Omnistar).*

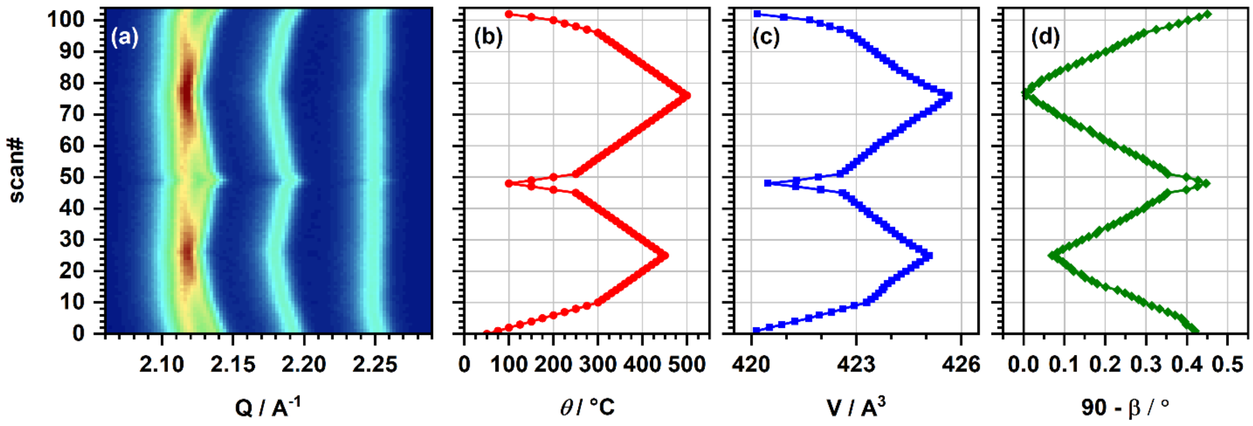

Figure S2: Contour plot for the main reflections of the *in situ* XRD patterns of $La_2Ni_{0.4}Cu_{0.6}O_3F_2$ obtained while heating and cooling the sample in a capillary open to air (a). The furnace temperature (b), refined unit cell volume (c) and reduced monoclinic angel (90 – β) (d) are additionally plotted.

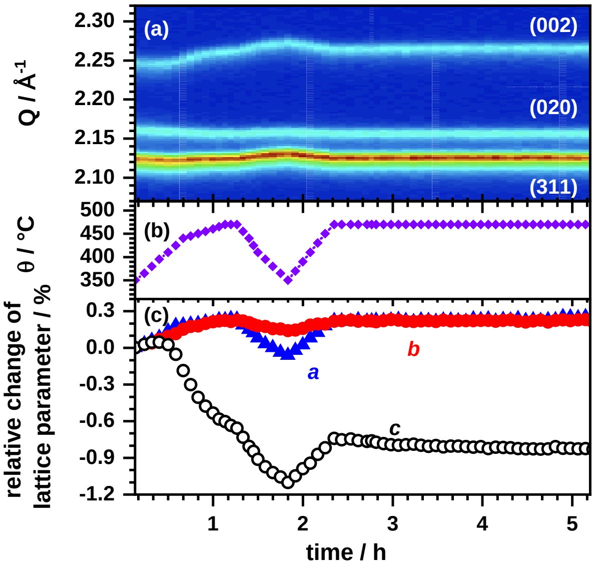

Figure S3: Detailed view of the main reflections ((311), (020) and (002)) of $La_2Ni_{0.8}Cu_{0.2}O_3F_2$ while heating (a). Sample temperature (b) and relative change of the lattice parameters (c) for subsequent XRD measurements taken at different temperatures. The decrease of parameter c in the first heating segment followed by the thermal increase in the second heating segment indicates an irreversible phase transition.

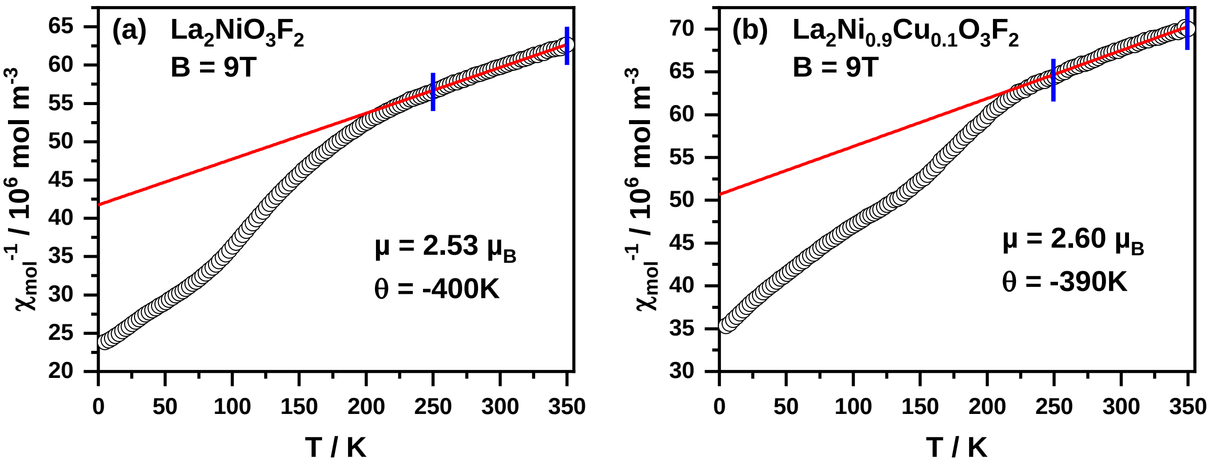

Figure S4: Inverse susceptibility vs. T plots for the oxyfluorides $La_2NiO_3F_2$ (a) and $La_2Ni_{0.9}Cu_{0.1}O_3F_2$ (b), demonstrating linear Curie Weiss behavior in the high temperature region.

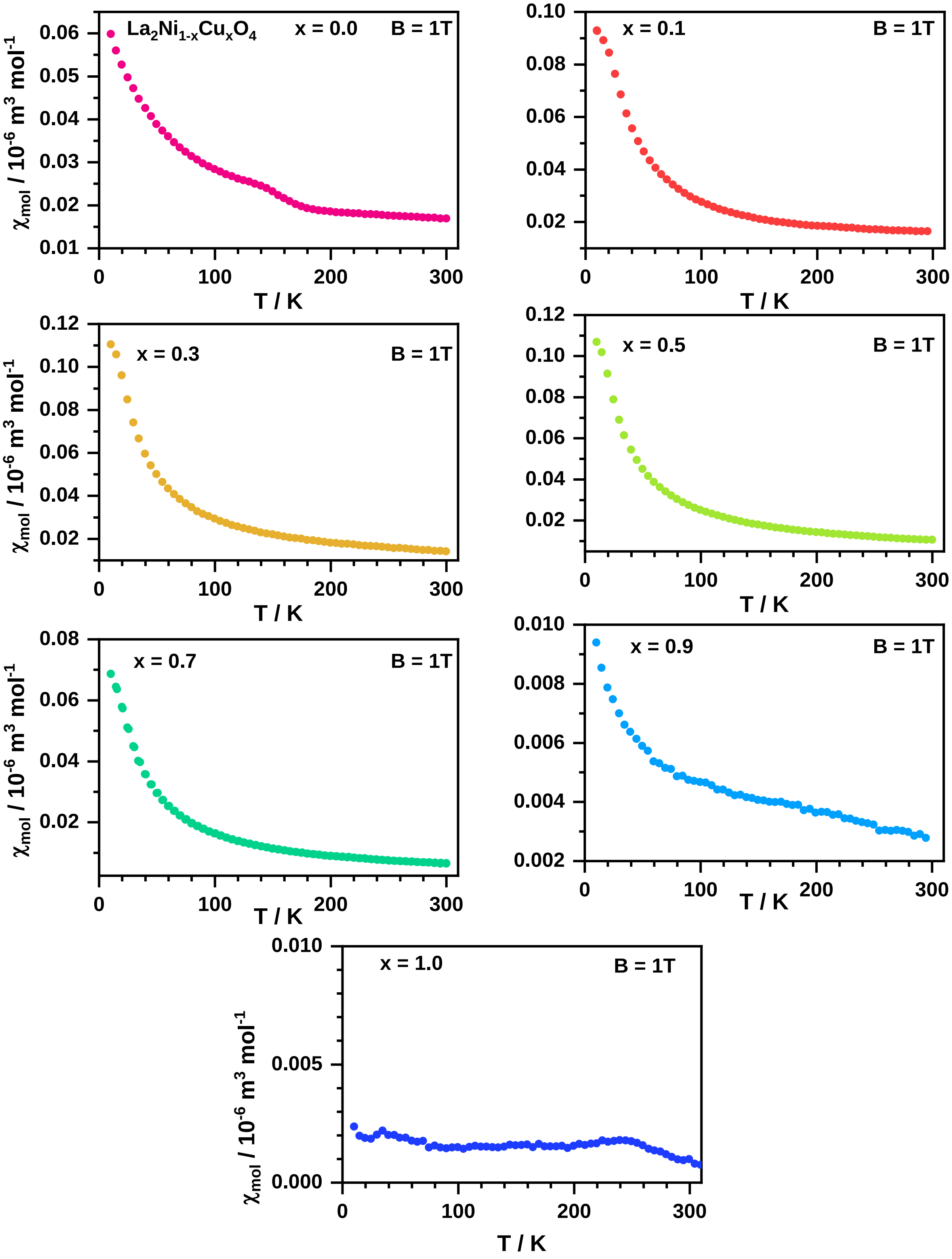

Figure S5: Molar susceptibility (χ_{mol}) versus temperature measurements of $La_2Ni_{1-x}Cu_xO_4$ (for x = 0, 0.1, 0.3, 0.5, 0.7, 0.9, 1) performed in an external field of 1T under field-cooled condition.
